# Retrospective Study on the Efficacy of Platelet-Rich Plasma Treatment in the Recovery of Quadriceps Muscle Strength After Anterior Cruciate Ligament Reconstruction in Non-Professional Athletes

**DOI:** 10.3390/jcm14103593

**Published:** 2025-05-21

**Authors:** Roxana Mihaela Munteanu, Bogdan Voicu, Diana Șandru, Arpad Solyom, Pia Simona Făgăraș, Tudor Sorin Pop

**Affiliations:** 1 Doctoral School of Medicine and Pharmacy, George Emil Palade University of Medicine, Pharmacy, Science and Technology of Târgu Mureș, 540142 Targu Mures, Romania; tudor.pop@umfst.ro; 2 OKF Medical Center Targu Mures, 540027 Targu Mures, Romania; drbogdanv@gmail.com (B.V.); sandrudiana58@yahoo.com (D.Ș.); arpad.solyom@umfst.ro (A.S.); 3 Faculty of Medicine, George Emil Palade University of Medicine, Pharmacy, Science and Technology of Târgu Mureș, 540142 Targu Mures, Romania; pia.fagaras@umfst.ro

**Keywords:** anterior cruciate ligament reconstruction, knee, muscle strength, quadriceps, platelet-rich plasma

## Abstract

**Background/Objectives**: This retrospective study aimed to evaluate whether PRP infiltrations improve quadriceps muscle strength recovery following anterior cruciate ligament reconstruction (ACLR), while minimizing the recovery time required to resume daily activities and sports. Numerous studies have explored the use of platelet-rich plasma (PRP) in treating ACL injuries. PRP therapy has demonstrated high efficacy in accelerating ligament healing in animal models. However, clinical trials involving human participants have reported inconsistent results regarding the effects of PRP on ACL reconstruction outcomes. **Methods**: Between 2020 and 2024, a total of 68 subjects who underwent ACLR were included in the study. Participants were divided into two groups, namely a treatment group that followed a standard rehabilitation protocol and received PRP infiltrations, and a control group that followed the same protocol without PRP treatment. Muscle strength was assessed using the isometric max strength balance (IMSB) test and the concentric max strength balance (CMSB) test, both performed using the Kineo Intelligent Load device (Globus Kineo 7000, Italian Excellence, Rome, Italy). **Results**: The results of IMSB test showed a significant difference between treatment groups according to a two-way ANOVA test (F(1, 198) = 7.345; *p* = 0.0073). The PRP-treated group showed significantly higher quadriceps muscle strength at 6 months (34.9 ± 9.6 vs. 30.0 ± 8.2 kg). The CMSB test also showed a significant difference at 6 months (F(1, 198) = 5.976; *p* = 0.00154), with the PRP-treated group having significantly higher concentric muscle strength (35.5 ± 9.5 vs. 30.7 ± 8.5 kg). **Conclusions**: These findings suggest that post-ligamentoplasty PRP infiltrations may have beneficial effects on muscle strength recovery. However, further prospective studies with larger sample sizes are necessary to confirm these results.

## 1. Introduction

The anterior cruciate ligament (ACL) is one of the two cruciate ligaments of the knee, playing a key role in stabilizing the joint. It is a strong band of connective tissue and collagen fibers originating from the anteromedial aspect of the intercondylar region of the tibial plateau and extending posterolaterally to attach to the medial aspect of the lateral femoral condyle. This attachment site includes two important anatomical landmarks, namely the lateral intercondylar ridge, which defines the anterior border of the ACL, and the bifurcate ridge, which separates the two bundles of the ACL. The ACL measures approximately 32 mm in length and 7 to 12 mm in width [1]. It is an extrasynovial structure, and fibroblasts are involved in its continuous renewal and maintenance [2]. The ACL also prevents anterior translation or rotation of the tibia relative to the femur and helps resist varus and valgus angulation forces [3].

Rupture of the ACL is a common injury, especially among young individuals and athletes, often caused by physical exertion [4]. Such injuries significantly reduce knee stability, impair athletic performance, increase the risk of subsequent meniscal damage, and may contribute to the early onset of knee joint degeneration [5]. Greater quadriceps strength and a smaller inter-limb strength deficit have consistently been associated with improved surgical outcomes [6,7]. Previous ACL reconstruction studies have shown a positive correlation between increased quadriceps strength and better knee function in the affected limb, as well as a reduced risk of recurrent injuries. Furthermore, impaired postoperative quadriceps strength has been identified as a potential risk factor for post-traumatic knee osteoarthritis and graft reinjury. Some studies also report higher return-to-sport rates following patellar tendon autografts compared to allografts in revision ACL reconstruction procedures.

The quadriceps femoris muscle contains predominantly anaerobic (white) fibers at the surface and more aerobic (red) fibers in the deeper layers. Anaerobic fibers are less vascularized than oxidative ones and are activated after red fibers. During contraction, the deeper fibers undergo slower deoxygenation. Males typically possess a higher percentage of contractile force and faster contraction speed than females, likely due to increased muscle mass and specific hormonal influences. Testosterone enhances motor unit firing rates, promotes greater and faster calcium release in muscle fibers (leading to faster contraction), and accelerates tissue repair and hypertrophic responses. Men also tend to have a higher proportion of white fibers. The quadriceps femoris acts on both the hip and knee joints: the rectus femoris contributes to hip flexion, while together with the vastus lateralis, vastus medialis, and vastus intermedius, it facilitates knee extension. Proper myoelectric balance within the quadriceps is crucial for patellar tracking, and proprioceptors within the muscle help maintain posture [8]. ACL injuries are not limited to athletes but also occur frequently among recreational athletes, middle-aged individuals, and even children. Platelet-rich plasma (PRP) therapy could be beneficial in these cases; however, its clinical applicability and limitations remain unclear, necessitating further studies [9]. PRP is a regenerative therapy that has gained popularity in musculoskeletal medicine due to its potential to enhance tissue repair in areas with low healing capacity [10]. It is an autologous blood product enriched with a platelet concentration higher than that found in normal blood, typically isolated via differential centrifugation [11]. PRP contains elevated levels of growth factors (GFs) [12], such as transforming growth factor-β, bone morphogenetic proteins, insulin-like growth factor, and platelet-derived growth factor. Intra-articular PRP injections create a microenvironment conducive to cell proliferation and tissue repair [13]. Animal studies have demonstrated PRP’s ability to promote tendon healing [14], although human clinical evidence remains limited [15]. While its role is still debated, PRP is believed to accelerate graft maturation and aid healing at both the bone tunnel and donor site. Theoretically, after ACL reconstruction, PRP delivers a rich mixture of growth factors and proteins into the joint environment, potentially accelerating regeneration. Platelets, being one of the primary sources of GFs, release bioactive molecules during fibrin clot formation. Among these, PDGF, TGF-β, IGF, and VEGF play key roles in musculoskeletal tissue repair by regulating chemotaxis, proliferation, differentiation, angiogenesis, and the clearance of cellular debris [16]. Additionally, PRP provides anabolic stimulation to cells, enhances extracellular matrix production, and exerts anti-inflammatory effects in the joint. Given its potential, PRP could offer multiple benefits for ACL reconstructive surgery, such as improved and faster graft integration, reduced early postoperative inflammation, enhanced incorporation of the graft into bone tunnels (minimizing tunnel enlargement and potential failure), and accelerated healing at the donor site [17].

The aim of this study was to identify the potential efficacy of PRP infiltrations for accelerating quadriceps strength recovery after ACL reconstruction. Although PRP has shown promising results in animal models, clinical trials involving human subjects have produced inconsistent outcomes.

To our knowledge, few retrospective clinical studies have specifically focused on quadriceps muscle strength recovery following ACL reconstruction using both isometric and concentric strength assessments with the Kineo Intelligent Load system in a standardized rehabilitation setting.

## 2. Materials and Methods

This retrospective study was approved by the Ethics Committee for Scientific Research of the George Emil Palade University of Medicine, Pharmacy, Science and Technology of Târgu Mureș (approval nr. 3562/20 January 2025). Prior to patient enrollment, pilot testing was conducted to standardize test protocols. Allocation to the PRP or control groups was not randomized and was based on surgeon discretion and patient preference at the time of surgery. Medical records of patients treated at OKF Medical Center in Târgu Mureș were studied. Between 2020 and 2024, 68 subjects who underwent an ACLR procedure were included in this study. Given the retrospective nature of the study, a formal a priori sample size calculation was not performed. However, a post hoc power analysis was conducted, revealing an effect size (Cohen’s d = 0.61) with a power of 0.82 at alpha = 0.05 for the primary outcome (quadriceps strength at 6 months). The patients were divided into two groups, namely a treated group that followed the recovery protocol and also received the platelet-rich plasma infiltrations and a control group that followed the recovery protocol without receiving the plasma infiltrations enriched with platelets. Controls were selected from the same clinical population and met identical inclusion criteria as the PRP group. Although no strict matching protocol was applied, groups were comparable in age, sex, graft type, and baseline quadriceps strength. Throughout the study, the data collected included patient age, body mass index (BMI), sex, type of graft used, general condition, and ongoing medication. Inclusion criteria required the existence of a series of quadriceps muscle strength tests—both isometric and isotonic—conducted at 2, 4, and 6 months postoperatively. To evaluate the effectiveness of orthobiologic treatment with PRP, the monitored parameters included muscle strength, strength deficit between the operated and non-operated limbs, and anthropometric data. Exclusion criteria were the presence of chronic cardiac or musculoskeletal diseases, use of systemic corticosteroid therapy, or occurrence of postoperative injuries during recovery. All subjects followed an identical recovery protocol, which included therapeutic physical exercises, respecting the established progression and recovery stages. The program included muscle activation, joint mobility, toning exercises, proprioception and motor control training, plyometric exercises, and sport-specific reconditioning, all under close supervision by licensed physiotherapists.

The muscular tests performed on patients in both groups were the isometric max strength balance test and the concentric max strength balance test, both performed on the Kineo Intelligent Load (Globus Kineo 7000 Italian Excellence, Rome, Italy). Prior to enrolling participants, pilot testing was conducted on a small sample (*n* = 5) to standardize testing angles and procedures for both IMSB and CMSB tests using the Kineo Intelligent Load system. Isometric Max Strength Balance testing was performed for each patient in 5 degrees of movement, at 30, 45, 60, 75, and 90 degrees, both for the affected limb and for the healthy limb; at the end of the testing, an average of the five repetitions performed. The concentric max strength balance test consisted of performing five knee extension movements performed with both the affected limb and with the healthy limb; at the end of the test an average of the muscle strength of each limb was also obtained, as well as an average of the existing force deficit. Before performing the muscle testing, the patients followed a program of warming up and preparing the body for effort, consisting of isotonic exercises for the muscles of the lower limbs, such as squats, lunges, and leg extensions. All patients followed the same warm-up schedule. To carry out the tests, the patients were positioned on the Kineo Intelligent Load, sitting on the device specially made for the knee extension movement, and they were instructed by the physiotherapist as what the respective test consisted of. The subjects’ knees were positioned at 90 degrees, and the force axis of the dynamometer was positioned just superior to the lateral malleolus of the ankle. During the isometric testing, the subjects were asked to perform a maximal contraction in each degree of movement, performing 5 maximal isometric repetitions in a row, first with the unaffected limb, then with the affected limb. Each contraction had an average duration of 5 s, followed by a 10 s pause between each of the 5 degrees where the tests were performed; between the 2 limbs, the patient had a 30 s pause.

During the isotonic testing, the subjects were asked to perform 5 knee extension movements, with explosive concentric contraction (when lifting) and a controlled return (eccentric, when lowering). First, they performed the 5 repetitions with the unaffected limb, then with the affected limb. Repetitions were performed without a rest between them, with subjects having a 30 s resting time between trials of the affected limb and trials of the unaffected limb. The results were analyzed for the strength of the quadriceps per concentric contraction, for each repetition separately, at the end of which the muscle strength on each limb was averaged, as well as the strength deficit average of the affected limb. The relative strength deficit was presented as a proportional difference compared to the uninjured limb. Positive values denoted a deficit in quadriceps strength in relation to the uninjured limb, whereas negative values indicated that the injured side exhibited greater strength than the uninjured limb.

Subjects in the experimental group received intra-articular PRP infiltrations, administered by the orthopedic surgeon approximately one week prior to each muscle test. PRP was prepared from the patients’ own blood, following the protocol described by Dhurat et al. [18]. Briefly, 30 mL of venous blood yielded approximately 3–5 mL of PRP, depending on the individual’s platelet count, the equipment, and the technique used. Blood collection involved an anticoagulant (citrate dextrose) to prevent premature platelet activation. Centrifugation was performed using a specialized centrifuge (XC SPINPLUS, Shanghai, China).

Statistical analysis was performed using GraphPad Prism 8 (GraphPad Software Inc.; San Diego, CA, USA). Significance levels were denoted as follows: * *p* < 0.05; ** *p* < 0.01; *** *p* < 0.001. The distribution of dichotomic type data was analyzed using a chi-square test. For assessing the differences between the control and PRP-treated groups, an unpaired *t*-test was performed. The repeated measure parameters were checked for outliers using Grubbs’ test and their distribution was analyzed by running a Kolmogorov–Smirnov test. The differences between different timepoints were assessed using a repeated measure ANOVA test. The limit for statistical significance was set to *p* < 0.05 in all analyses. To evaluate the influence of the PRP treatment on muscle strength along with the registered covariables, such as age, sex, and tendon type, a mixed linear regression analysis was carried out using Python (version 3.10.2) with the following libraries: pandas, pymer4, and statsmodel [19]. The limit for statistical significance was set to *p* < 0.05 in all analyses. Due to the exploratory nature of the analysis and the limited number of comparisons, *p*-values were interpreted without formal adjustment for multiple testing. This approach was deemed appropriate to avoid excessive type II errors in a hypothesis-generating context.

## 3. Results

A total of 68 patients were enrolled in this study, of which 34 were treated with PRP infiltrations. The treated group (74% male, mean ± SD age 30.1 ± 8.6) showed the same demographical characteristics as the control group (74% male, mean ± SD age 31.3 ± 8.6). Moreover, the graft source showed the same distribution pattern (hamstring/pattelar 30/4 vs. 31/3) (Table 1). All patients were checked for specific metabolic diseases, such as diabetes, autoimmune diseases, dyslipidemias, and specific drug treatments. Bodyweight and height were recorded, but due to the lack of complete information these parameters were not included in the statistical analysis.

Quadriceps muscle strength was determined for both limbs and the recovery process was characterized by the increase in affected limb muscle strength and the decrease in the deficit between the two limbs.

One of the most important objective of the recovery protocol after ACLR is to achieve a deficit of quadriceps muscle strength below 10%. The number of subjects in the control group that achieved this objective was 22 of 34 (64.7%), whereas in the PRP-treated group there were 30 of 34 subjects (88.2%). This difference was significant according to Fisher’s exact test (*p* = 0.00433).

**Figure 1:**
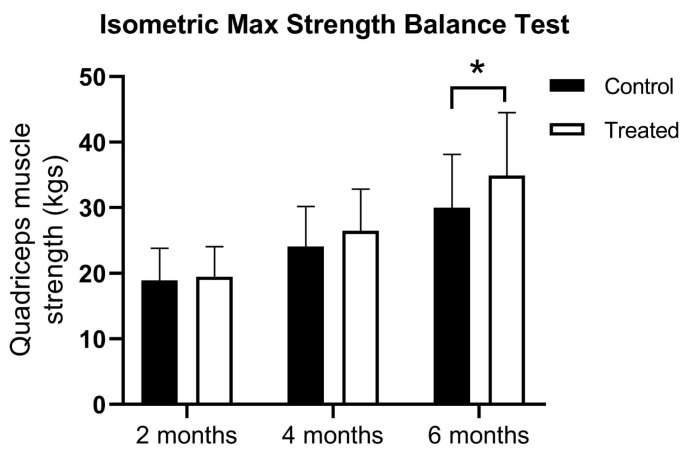
The results of the isometric max strength balance test at 2, 4, and 6 months after ACLR. Data are expressed as mean ± standard deviation; * *p* < 0.05 vs. control.

At the first muscle testing there were no difference between the groups using the isometric max strength balance test (18.91 ± 4.91 vs. 19.43 ± 4.63 kg). At 4 months, there was a higher muscle strength registered for PRP patients, but the statistical significance limit was not reached (24.06 ± 6.11 vs. 26.43 ± 6.40 kg). However, the PRP-treated group showed significantly higher quadriceps muscle strength at 6 months (30.0 ± 8.2 vs. 34.9 ± 9.6 kg) and showed a significant difference between treatment groups according to a two-way ANOVA test (F(1, 198) = 7.345; *p* = 0.0073) (Figure 1).

**Figure 2:**
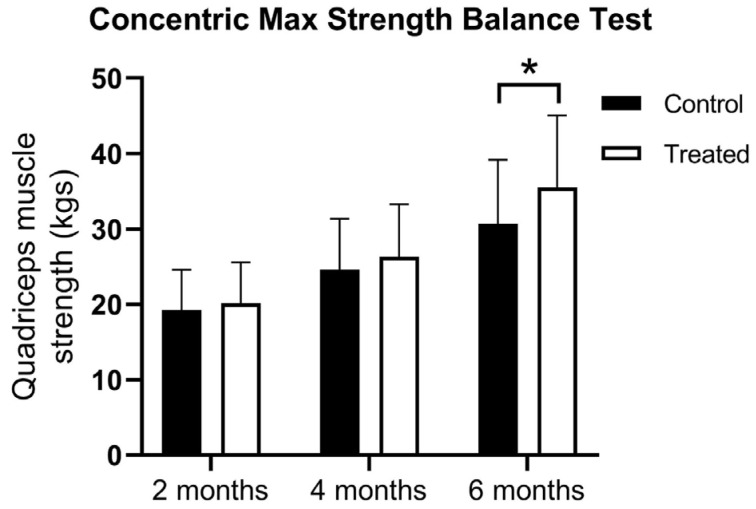
The results of the concentric max strength balance test at 2, 4, and 6 months after ACLR. Data are expressed as mean ± standard deviation; * *p* < 0.05 vs. control.

At the first muscle testing, there were no difference between the groups using the concentric max strength balance test (19.28 ± 5.32 vs. 20.15 ± 5.43 kg). At 4 months, there was higher muscle strength in the PRP-treated group, but the statistical significance limit was not reached (24.62 ± 6.75 vs. 26.33 ± 6.95 kg). However, at 6 months, the PRP-treated group showed significantly higher muscle strength (30.7 ± 8.5 vs. 35.5 ± 9.5 kg) according to the two-way ANOVA Test (F(1, 198) = 5.976; *p* = 0.00154) (Figure 2).

**Figure 3:**
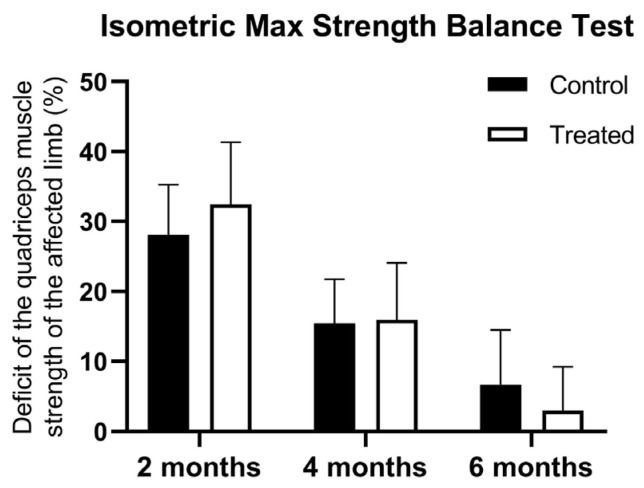
Muscle deficit of the quadriceps on the isometric max strength balance test at 2, 4, and 6 months after ACLR. Data are expressed as mean ± standard deviation.

At the first muscle testing there were no differences between the groups using the isometric max strength balance Test in terms of strength of the quadriceps between the injured and non-injured limb (28.07 ± 7.19 vs. 32.43 ± 8.91 kg). At 4 months, there was a higher muscle deficit registered for control patients, but the statistical significance limit was not reached (15.42 ± 6.32 vs. 15.95 ± 8.11 kg). However, the PRP-treated group showed a lower quadriceps muscle strength deficit at 6 months (6.68 ± 7.85 vs. 3.04 ± 6.20 kg) and showed a significant interaction between treatment and time points (F(2, 198) = 4.853, *p* = 0.0088) (Figure 3).

**Figure 4:**
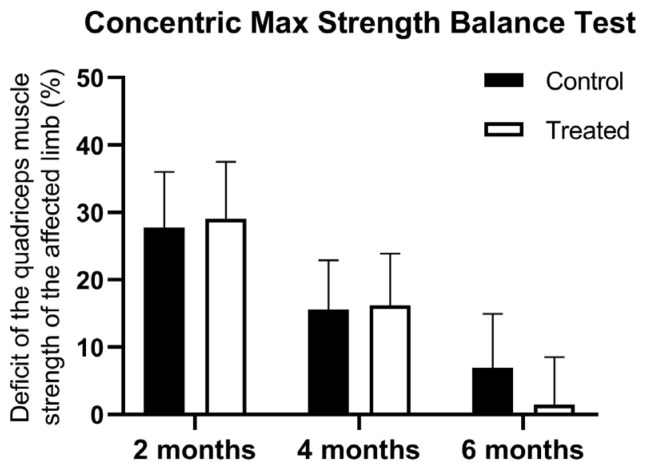
Muscle deficit of the quadriceps on concentric max strength balance test at 2, 4, and 6 months after ACLR. Data are expressed as mean ± standard deviation.

At the first muscle testing, there were no difference between the groups using the concentric max strength balance test in terms of the strength of the quadriceps between the injured and non-injured limb (27.73 ± 8.28 vs. 29.05 ± 8.45 kg). At 4 months, there was a higher muscle deficit registered for control patients, but the statistical significance limit was not reached (15.56 ± 7.35 vs. 16.22 ± 7.69 kg). However, the PRP-treated group showed significantly lower quadriceps muscle strength deficit at 6 months (6.89 ± 8.03 vs. 1.47 ± 7.06 kg) and showed a significant interaction between treatment and time points (F(2, 198) = 3.839, *p* = 0.0231) (Figure 4).

For each dependent variable (i.e., isometric and concentric muscle strength, relative strength deficit) the mixed linear regression models were constructed by taking into account all independent variables (age, sex, graft type, and treatment) and the three time points of the strength determinations. The interactions between independent variables were also considered. Several models were tested, and based on the AIC values, the best fit model was chosen (Table 2). The results obtained for each dependent variable are presented in Table 3 and Table 4. Briefly, both isometric and concentric strength deficits were significantly influenced by treatment, time, and treatment × time interaction. Bootstrap analysis with 1000 resamples was performed for isometric and concentric strength outcomes. The bootstrapped 95% confidence intervals corroborated the initial estimates, reinforcing their validity.

## 4. Discussion

During our study, we observed that PRP infiltrations can enhance quadriceps muscle strength following ACL reconstruction (ACLR) and reduce the strength deficit between the injured and uninjured limb. This improvement may result from pain reduction, which allows patients to adhere more effectively to the recovery program and even tolerate increased muscular load during rehabilitation. Another possible explanation is that PRP promotes graft healing and integration, enabling a smoother rehabilitation process and boosting patient confidence and optimism throughout recovery.

It is well documented that significant quadriceps atrophy and weakness occur following knee joint injuries. Such trauma induces arthrogenic muscle inhibition (AMI)—a presynaptic reflex inhibition of the musculature surrounding the injured joint. AMI is known to persist both after ACL injury and during the rehabilitation process following ACL reconstruction; however, the exact mechanisms behind AMI and their full impact are still being investigated. It is also well established that central neural activation deficits persist after ACL reconstruction, potentially impairing muscle recruitment [20].

The exact causes of persistent quadriceps atrophy post-ACLR remain unclear, as noted in the study by Tim-Yun Ong et al. [21]. Increasing research interest has been directed toward understanding the trophic mechanisms through which exercise stimulates muscle hypertrophy, and why certain patients exhibit an insufficient muscle growth response during rehabilitation. One potential explanation lies in the dysregulation of myokine production and release in response to resistance training [21].

Quadriceps strength deficits are common following ACL reconstruction. Despite structured rehabilitation programs, these deficits often persist, delaying the return to daily activities and athletic performance [22]. Achieving symmetrical quadriceps strength, defined as equal strength between the injured and uninjured limb, is a crucial goal of rehabilitation. Greater limb asymmetry after ACLR has been associated with failure to meet return-to-sport criteria, poorer functional outcomes, and altered lower limb biomechanics during gait. Even more concerning, quadriceps weakness following ACL injury has been linked to the long-term risk of developing early-onset post-traumatic osteoarthritis [22].

As shown in the study by Palmieri-Smith et al. [22], given the detrimental effects of quadriceps weakness, it is essential to maximize quadriceps strength in order to preserve knee joint health and functional capacity. In that study, quadriceps strength was measured isometrically, similar to our methodology, using a dynamometer. Isokinetic quadriceps muscle strength was also assessed bilaterally. Briefly, participants were positioned on a dynamometer with the test limb’s knee and hip flexed to 90°. They performed three voluntary concentric knee extensions at 60°/s, with two-minute rest intervals between repetitions. Verbal encouragement and real-time visual feedback were provided throughout the assessment. The quadriceps index (QI) was then calculated to classify participants into three groups, namely the HIGH group: QI ≥ 90%, MOD group: QI < 90% but ≥ 80%, LOW group: QI < 80%. Strength asymmetries of ≤10% are typically considered acceptable for return to activity after ACLR and were, thus, used to define the HIGH group. Asymmetries of ≥20% were classified as abnormal and used to define the LOW group [22].

In the study by Schwery et al. [23], which also used isometric testing, quadriceps strength was assessed following ACL reconstruction. Their findings showed that an isometric testing protocol using an isokinetic dynamometer is a safe, reliable, and clinically reasonable method for evaluating quadriceps strength in the early stages of rehabilitation.

In the study by Czamara et al. [24], both isometric and isokinetic strength tests were applied. The results showed that, by the 17th week of physical therapy, significantly lower extensor and flexor torque values were recorded in the operated knee compared to the uninjured knee for both types of tests. Similarly, in our study, certain subjects—particularly those in the control group—demonstrated continued muscle deficits during the same period.

In the study by Kyriakidou et al. [25], the maximal isometric force of the affected limb was evaluated using the Kineo dynamometer (Globus Kineo 7000, Italy), as in our study. Participants were seated upright and secured to the dynamometer to minimize compensatory movement. The chair was adjusted so that the footrest was placed under the tibialis anterior, and the pivot aligned with the lateral epicondyle of the dominant leg. In contrast to our protocol, which involved measurements at five angles (30°, 45°, 60°, 75°, and 90°), their study assessed maximum force at a single angle of 60°. The protocol consisted of three maximal isometric contractions, each lasting 3–5 s, with 120 s of rest between repetitions. After a 2-min rest period, participants applied maximal isometric force against the footrest. The peak force was calculated as the average of the three repetitions. From pilot data (*n* = 6 healthy young participants), the within-day coefficient of variation (CV) for leg extension MVIC was found to be 6.2%, while the day-to-day CV was 8.7%. Verbal encouragement was provided during each repetition, just as it was in our study.

Given the ubiquity of quadriceps muscle weakness following anterior cruciate ligament (ACL) injury and surgery, restoring quadriceps strength remains a key goal in rehabilitation protocols for individuals undergoing ACL reconstruction (ACLR) [26,27]. Recovery is commonly assessed by comparing the strength of the affected limb to that of the uninjured limb—a metric referred to as the limb symmetry index (LSI) [28,29]. Achieving a predetermined LSI threshold, typically 90%, is often used as a benchmark for readiness to return to sport [30]. Although current guidelines advocate using the LSI as a return-to-play criterion, research suggests that the LSI may overestimate actual recovery of quadriceps function, especially in high-performance individuals [31]. Therefore, it is essential to establish more objective and comprehensive measures for evaluating quadriceps strength recovery in this patient population.

Our study suggests that PRP infiltrations had a beneficial effect on quadriceps muscle strength recovery following ACLR. This aligns with findings by Lv et al. [32], who observed the following significant differences between PRP-treated and control groups in several outcome measures at six months postoperatively: VAS score: mean difference (MD) −1.12, 95% CI [−1.92, −0.31]; *p* = 0.007, subjective IKDC score: MD 6.08, 95% CI [4.39, 7.77]; *p* < 0.00001, and Lysholm score: MD 8.49, 95% CI [1.63, 15.36]; *p* = 0.02. However, only the reduction in pain was deemed clinically significant. By the time of the one-year follow-up, no clinically meaningful improvements were observed in VAS score: MD −0.47; *p* = 0.04, subjective IKDC score: MD 3.99; *p* =0.03, Lysholm score: MD 2.30; *p* = 0.32, objective IKDC score: risk ratio (RR) 1.03; *p* =0.09, or knee joint laxity: MD 0.17; *p* = 0.28. In contrast, the study by McRobb et al. [33] found early improvements in ligamentization, vascularization, and pain reduction, along with a decrease in inflammation. However, no significant effects were noted during later stages of the healing process, and no consensus was reached regarding long-term benefits.

Compared to the existing literature, our study provides a distinct methodological contribution by integrating concentric and isometric strength testing using the Kineo system, under a unified rehabilitation protocol in a non-professional athlete population.

This study has a number of limitations, being a retrospective study, limited by a relatively small number of subjects and non-existent data on the diet and nutritional supplements of the subjects chosen, which could have influenced their recovery, as well as an unequal distribution between males and females, which may be representative, especially for athletes. We were also missing data on subjects’ body mass index. This retrospective assignment may introduce selection bias and should be considered when interpreting the study results.

The mixed linear models used in this study are exploratory and should be interpreted with caution due to the limited sample size. While AIC-based model selection indicated an acceptable fit, further validation in larger prospective cohorts is warranted.

## 5. Conclusions

The administration of orthobiologic treatment with PRP at 2, 4, and 6 months after anterior cruciate ligament reconstruction helped regain quadriceps muscle strength, demonstrated in isotonic and isometric tests performed during the recovery period.

Based on these results, post-ligamentoplasty PRP infiltrations might have beneficial effects in regaining muscle strength, but prospective studies on a larger sample are needed in the future.

## Figures and Tables

**Table 1:** Baseline characteristics of the control and treated groups.

Parameter	Control Group *n* = 34	Treated Group *n* = 34	Statistical Significance
Age	31.3 ± 1.5	30.1 ± 1.5	*p* = 0.564
Gender (m/f)	25/9	25/9	*p* = 1
BMI	23.8 ± 0.7	24.8 ± 0.6	*p* = 0.382
Graft (hamstring/patellar)	30/4	31/3	*p* = 0.689

**Table 2:** Comparison of different mixed linear regression models examining the effects of several independent variables and covariates on isometric and concentric strength deficits.

Dependent Variable/Model	Independent Variables and Covariates Included ^1^	Akaike Information Criterion (AIC)
Isometric strength deficit/1	Age, sex, treatment	−215.216
Isometric strength deficit/2	Age, sex, graft type, treatment	−208.303
Isometric strength deficit/3	Age, sex, graft type, treatment, timepoint	−428.252
Isometric strength deficit/4	Age, sex, graft type, treatment × timepoint	−433.395
Isometric strength deficit/5	Age, sex × timepoint, graft type, treatment	−426.690
Isometric strength deficit/6	Age, sex × treatment, graft type, timepoint	−422.116
Concentric strength deficit/1	Age, sex, treatment	−226.896
Concentric strength deficit/2	Age, sex, graft type, treatment	−220.023
Concentric strength deficit/3	Age, sex, graft type, treatment, timepoint	−423.879
Concentric strength deficit/4	Age, sex, graft type, treatment × timepoint	−424.292
Concentric strength deficit/5	Age, sex × timepoint, graft type, treatment	−416.570
Concentric strength deficit/6	Age, sex × treatment, graft type, timepoint	−416.967

^1^ Independent variables were defined as follows: age (continuous), sex (female = 0; male = 1), graft type (patellar tendon = 0; hamstring tendon = 1), and treatment (no injection = 0; PRP injection = 1).

**Table 3:** Results of the mixed linear model regression analysis of the isometric strength deficit.

Parameter	Estimate	2.5% CI	97.5% CI	SE	DF	T-Stat	*p*-Value	Significance
Intercept	0.403	0.307	0.500	0.049	75.348	8.210	<0.0001	***
Age	−0.001	−0.003	0.001	0.001	63.000	0.968	0.337	
Sex	0.012	−0.021	0.046	0.017	63.000	0.730	0.468	
Graft type	−0.001	−0.053	0.050	0.026	63.000	−0.052	0.958	
Treatment	0.083	0.034	0.132	0.025	197.000	3.331	<0.0001	**
Timepoint	−0.107	−0.121	−0.093	0.007	134.000	−14.710	<0.0001	***
Treatment/timepoint	−0.040	−0.060	−0.020	0.010	134.000	−3.893	<0.0001	***

Independent variables were defined as follows: age (continuous), sex (female = 0; male = 1), graft type (patellar tendon = 0; hamstring tendon = 1), and treatment (no injection = 0; PRP injection = 1). Abbreviations: CI = confidence interval, SE = standard error, and DF = degree of freedom. ** *p* < 0.01; *** *p* < 0.001.

**Table 4:** Results of the mixed linear model regression analysis of the concentric strength deficit.

Parameter	Estimate	2.5% CI	97.5% CI	SE	DF	T-Stat	*p*-Value	Significance
Intercept	0.422	0.326	0.517	0.049	76.879	8.672	<0.0001	***
Age	−0.001	−0.003	0.001	0.001	63.000	−1.036	0.304	
Sex	−0.032	−0.065	0.001	0.017	63.000	−1.900	0.062	
Graft type	0.011	−0.040	0.061	0.026	63.000	0.404	0.688	
Treatment	0.055	0.005	0.105	0.026	196.452	2.137	0.034	*
Timepoint	−0.104	−0.119	−0.089	0.008	134.000	−13.749	<0.0001	***
Treatment/timepoint	−0.034	−0.055	−0.013	0.011	134.000	−3.151	0.002	**

Independent variables were defined as follows: age (continuous), sex (female = 0; male = 1), graft type (patellar tendon = 0; hamstring tendon = 1), and treatment (no injection = 0; PRP injection = 1). Abbreviations: CI = confidence interval, SE = standard error, DF = degree of freedom. * *p* < 0.05; ** *p* < 0.01; *** *p* < 0.001.

## Data Availability

The data presented in this study are available on request from the corresponding author.
